# Community‐based differentiated service delivery models incorporating multi‐month dispensing of antiretroviral treatment for newly stable people living with HIV receiving single annual clinical visits: a pooled analysis of two cluster‐randomized trials in southern Africa

**DOI:** 10.1002/jia2.25819

**Published:** 2021-10-28

**Authors:** Geoffrey Fatti, Nicoletta Ngorima‐Mabhena, Appolinaire Tiam, Betty Bawuba Tukei, Tonderai Kasu, Trish Muzenda, Khotso Maile, Carl Lombard, Charles Chasela, Ashraf Grimwood

**Affiliations:** ^1^ Kheth'Impilo AIDS Free Living Cape Town South Africa; ^2^ Division of Epidemiology and Biostatistics Department of Global Health Faculty of Medicine and Health Sciences Stellenbosch University Cape Town South Africa; ^3^ Elizabeth Glaser Pediatric AIDS Foundation Washington DC USA; ^4^ Right to Care/EQUIP Health Maseru Lesotho; ^5^ Ministry of Health and Child Care Harare Zimbabwe; ^6^ Division of Public Health Medicine School of Public Health and Family Medicine University of Cape Town Cape Town South Africa; ^7^ Biostatistics Unit South African Medical Research Council Cape Town South Africa; ^8^ Right to Care/EQUIP Health Centurion South Africa; ^9^ Department of Epidemiology and Biostatistics School of Public Health Faculty of Health Sciences University of the Witwatersrand Johannesburg South Africa

**Keywords:** antiretroviral treatment, cluster‐randomized trial, COVID‐19, differentiated service delivery, multi‐month dispensing, operational research

## Abstract

**Introduction:**

Differentiated service delivery (DSD) models for HIV treatment decrease health facility visit frequency and limit healthcare facility‐based exposure to severe acute respiratory syndrome coronavirus 2. However, two important evidence gaps include understanding DSD effectiveness amongst clients commencing DSD within 12 months of antiretroviral treatment (ART) initiation and amongst clients receiving only single annual clinical consultations. To investigate these, we pooled data from two cluster‐randomized trials investigating community‐based DSD in Zimbabwe and Lesotho.

**Methods:**

Individual‐level participant data of newly stable adults enrolled between 6 and 12 months after ART initiation were pooled. Both trials (conducted between August 2017 and July 2019) had three arms: Standard‐of‐care three‐monthly ART provision at healthcare facilities (SoC, control); ART provided three‐monthly in community ART groups (CAGs) (3MC) and ART provided six‐monthly in either CAGs or at community‐distribution points (6MC). Clinical visits were three‐monthly in SoC and annually in intervention arms. The primary outcome was retention in care and secondary outcomes were viral suppression (VS) and number of unscheduled facility visits 12 months after enrolment. Individual‐level regression analyses were conducted by intention‐to‐treat specifying for clustering and adjusted for country.

**Results and Discussion:**

A total of 599 participants were included; 212 (35.4%), 128 (21.4%) and 259 (43.2%) in SoC, 3MC and 6MC, respectively. Few participants aged <25 years were included (n = 32). After 12 months, 198 (93.4%), 123 (96.1%) and 248 (95.8%) were retained in SoC, 3MC and 6MC, respectively. Retention in 3MC was superior versus SoC, adjusted risk difference (aRD) = 4.6% (95% CI: 0.7%−8.5%). Retention in 6MC was non‐inferior versus SoC, aRD = 1.7% (95% CI: −2.5%−5.9%) (prespecified non‐inferiority aRD margin −3.25%). VS was similar between arms, 99.3, 98.6 and 98.1% in SoC, 3MC and 6MC, respectively. Adjusted risk ratio's for VS were 0.98 (95% CI: 0.92−1.03) for 3MC versus SoC, and 0.98 (CI: 0.95−1.00) for 6MC versus SoC. Unscheduled clinic visits were not increased in intervention arms: incidence rate ratio = 0.53 (CI: 0.16−1.80) for 3MC versus SoC; and 0.82 (CI: 0.25−2.79) for 6MC versus SoC.

**Conclusions:**

Community‐based DSD incorporating three‐ and six‐monthly ART refills and single annual clinical visits were at least non‐inferior to standard facility‐based care amongst newly stable ART clients aged ≥25 years.

ClinicalTrials.gov: NCT03238846 & NCT03438370

## 1 INTRODUCTION

Multi‐month dispensing (MMD) of antiretroviral treatment (ART) is a component of a number of differentiated service delivery (DSD) models that extends the period between ART refills to three‐ or six‐monthly [1]. MMD increases the efficiency of overburdened health systems in resource‐limited settings and is preferred by ART clients as the burden and costs of frequent facility visits are reduced [2, 3]. In the COVID‐19 era, reducing facility visit frequency and enabling ART receipt outside of health facilities are crucial DSD adaptations to safeguard both ART clients and healthcare workers from severe acute respiratory syndrome coronavirus 2 (SARS‐CoV‐2) infection [4, 5]. Safely scaling‐up DSD to as great a number of ART clients as possible in resource‐limited settings with high HIV prevalence is an urgent priority for health systems facing both pandemics of HIV and COVID‐19 [4].

DSD models incorporating MMD have recently been found to be non‐inferior to standard‐of‐care ART provision in three cluster‐randomized trials (CRTs) in southern Africa [6, 7, 8]. However, in these and other studies, participants received ART for prolonged time periods before commencing DSD (up to median 7 years) with very few who initiated DSD within 12 months of ART initiation [7, 9, 10, 11]. Without empirical evidence being available, it is currently unclear whether the safety and effectiveness of DSD is generalizable to newly stable clients within 12 months of ART initiation [12]. In some countries, eligibility to receive DSD and MMD has been reduced to 6 months from ART initiation; however, MMD eligibility remains at 12 months after ART initiation according to national policy in many sub‐Saharan African countries and India [13]. Defining these eligibility criteria has important consequences for ART clients, noting that inadequate time since ART initiation was the most frequent reason for ineligibility for MMD in a recent study from Zambia and Malawi [14].

Regarding the frequency of clinical visits, the World Health Organization (WHO) currently recommends that clinical visits be offered three‐ to six‐monthly for people established on ART [15]. Some countries have, however, reduced health facility visit frequency to only once annually (including in a CRT from South Africa [7]), which limits potential SARS‐CoV‐2 exposure and reduces burdens and costs for health systems and ART clients [16]. However, little randomized evidence regarding the safety and effectiveness of single annual clinical visits for newly stable ART clients is available. To investigate the effectiveness of community‐based DSD for ART clients initiating DSD specifically within 12 months of ART initiation with single annual facility visits, we pooled data from two large operational research CRTs investigating DSD to increase the sample of newly stable participants.

## 2 METHODS

Individual‐level participant data (IPD) from two CRTs in Zimbabwe and Lesotho were pooled. The aim of both trials was to assess whether community‐based DSD models incorporating MMD are non‐inferior to standard‐of‐care facility‐based ART provision for stable ART patients. The trials were conceptualized and implemented concurrently, had similar protocols, similar inclusion criteria, similar intervention and control arms, and similar hypotheses and outcomes, thus, data from the trials were suitable for pooling. The trials are described in detail elsewhere [6, 8, 17, 18]. Briefly, both trials were three‐arm, parallel, unblinded, pragmatic, non‐inferiority CRTs. Each arm in both trials consisted of ten health facilities (clusters) as follows:

- **Control arm (SoC)**: Participants received standard‐of‐care ART and clinical consultations at three‐monthly intervals at facilities.
- **Intervention arm 1 (3MC)**: Participants received ART at three‐monthly intervals in community ART groups (CAGs) with annual facility visits and clinical consultations.
- **Intervention arm 2 (6MC)**: Participants received ART at six‐monthly intervals in CAGs (Zimbabwe) or community distribution points (Lesotho) with annual facility visits and clinical consultations.

Study facilities (n = 60) were public health facilities in eight districts of the two countries. Clusters were allocated to the arms in each country with randomization stratified by urban/rural location and hospital/primary healthcare clinic. Adults (≥18 years) were eligible for enrolment if they were stable on ART, defined as receiving standard first‐line ART for ≥6 months and having a suppressed viral load (VL) (<1000 copies/mL) within the last 12 months, without active opportunistic infections or comorbidities requiring facility visits more frequently than six‐monthly, and who were not pregnant or postpartum. Recruitment commenced in August 2017 and follow‐up was completed in July 2019. In Zimbabwe and Lesotho, national ART guidelines had recently been modified to allow ART clients to be eligible for DSD from 6 months after ART initiation, which differed from the prevailing WHO guidelines which recommended DSD eligibility from 12 months after ART initiation [19]. As we were specifically interested in outcomes amongst those who enrolled ≤12 months following ART initiation, analyses were restricted to those who initiated ART between 6 and 12 months previously.

The model of care for each arm is given in detail in Table S1. After 12 months, all participants were scheduled to receive a clinical consultation, VL testing and ART supply at the facility, where VL results were reported as unsuppressed, patients were recalled to the clinics. The trials were embedded in routine healthcare services with no interference by study staff in the healthcare models.

The primary outcome was the proportion remaining in ART care 12 months after enrolment by intention‐to‐treat including participants in each arm as per baseline allocation. Retention in care is a critical indicator of ART program success [20]. The principal hypothesis was that retention for both intervention arms would be non‐inferior versus control (SoC) with a non‐inferiority margin of −3.25% (risk difference [RD]), as per the original trials. Secondary outcomes were proportions achieving viral suppression (VS) after 12 months, and the number of unscheduled facility visits between months 0 and 12. As VL testing infrastructure scale‐up was incomplete in these countries during the study, VS was a secondary outcome and we used participants with available VL results as the denominator for VS analyses.

Retention in care was defined as one‐participant attrition, where attrition was defined as either death (all‐cause) or loss to follow‐up (LTFU). LTFU was defined as no ART collection for >90 days after the last missed scheduled ART collection date. Participants not arriving for the scheduled 12‐month visit were considered retained if collecting ART within 90 days following the appointment date. Participants transferring‐out were censored at the date of transfer. VS was defined as VL <1000 copies/mL. Those eligible for outcome VL testing were enrolled participants excluding those who died, were lost to‐follow‐up or who had transferred‐out. Unscheduled facility visits were defined as any visit to the study clinics for any reason outside of visits scheduled by the assigned model of care.

For the main outcomes analyses, we performed “one‐stage” IPD meta‐analyses (stratified by trial), being appropriate when few trials are included, when participant numbers are small or when outcome events are rare [21, 22, 23, 24]. These analyses are detailed in the Supporting information. As an additional analysis for the primary outcome, a “two‐stage” meta‐analysis of IPD was performed by estimating cluster‐adjusted RDs separately for each trial and then combining these to estimate pooled RDs using random‐effects meta‐analysis. Heterogeneity was assessed using the *I* ^2^ statistic and forest plots. Ethical approval was provided by the Stellenbosch University Health Research Ethics Committee, reference S20/05/128.

## 3 RESULTS AND DISCUSSION

Data of 5336 participants from Lesotho and 4800 from Zimbabwe were pooled (total of 10,136 participants) (Figure S1). Amongst these, 9537 were enrolled >12 months after ART initiation and excluded. Thus, 599 participants enrolled between 6 and 12 months after ART initiation were included; 212 (35.4%), 128 (21.4%) and 259 (43.2%) in arms SoC, 3MC and 6MC, respectively. Baseline clinical variables were similar between arms. Little variation between arms was apparent regarding time from ART initiation until study enrolment (Table 1). Few participants aged <25 years were included (n = 32).

**Table 1:** Characteristics of participants at enrolment according to study arm

	SoC (control) (n = 212)	3MC (n = 128)	6MC (n = 259)	All participants (n = 599)
Age (years), median (IQR)	38.6 (32.2–48.1)	42.6 (35.7–50.7)	39.8 (32.1–49.6)	39.8 (32.8–49.6)
Age categories, n (%)				
18–24 years	15 (7.1)	4 (3.1)	13 (5.0)	32 (5.3)
25–49 years	151 (71.2)	91 (71.1)	189 (73.0)	431 (72.0)
≥ 50 years	46 (21.7)	33 (25.8)	57 (22.0)	136 (22.7)
Female, n (%)	118 (55.7)	96 (75.0)	167 (64.5)	381 (63.6)
Duration from ART initiation to study enrolment, months, median (IQR)	10.5 (8.9–11.6)	9.8 (8.2–11.3)	10.5 (9.1–11.5)	10.4 (8.7–11.5)
Time from HIV diagnosis to ART initiation, months, median (IQR)	0 (0–1.7)	0 (0–20.2)	0 (0–5.5)	0 (0–2)
WHO clinical stage				
Stage I or II	184 (86.8)	105 (82.0)	206 (79.5)	495 (82.6)
Stage III	23 (10.9)	19 (14.8)	51 (19.7)	93 (15.5)
Not recorded	5 (2.4)	4 (3.1)	2 (0.8)	11 (1.8)
CD4 cell count, cells/μL, median (IQR)	485 (289–654)	460.5 (310–716)	513.5 (318–640)	486 (306–654)
Weight, kg, median (IQR)	60.8 (55–67)	62 (54.7–74.9)	60.8 (54–70)	61 (54.3–69.8)
Year of ART initiation, median (IQR)	2016 (2016–2017)	2017 (2016–2017)	2017 (2016–2017)	2017 (2016–2017)
Disclosed HIV status, n (%)	200 (94.3)	119 (93.0)	246 (95.0)	565 (94.5)
Unemployed, n (%)	123 (58.0)	77 (60.2)	125 (48.3)	325 (54.4)
Married, n (%)	121 (57.1)	61 (47.7)	154 (59.5)	336 (56.2)
Currently drinks alcohol, n (%)	47 (22.2)	18 (14.1)	48 (18.5)	113 (18.9)
Facility type				
Primary healthcare clinic, n (%)	151 (71.2)	108 (84.3)	181 (69.9)	440 (73.5)
Hospital‐based facility, n (%)	61 (28.8)	20 (15.6)	78 (30.1)	159 (26.5)
Location				
Rural, n (%)	153 (72.2)	70 (54.7)	211 (81.5)	434 (72.5)
Urban, n (%)	59 (27.8)	58 (45.3)	48 (18.5)	165 (27.5)
Country				
Lesotho, n (%)	118 (55.7)	51 (39.8)	150 (57.9)	319 (53.3)
Zimbabwe, n (%)	94 (44.3)	77 (60.2)	109 (42.1)	280 (46.7)

SoC‐participants received three‐monthly dispensing of ART at the facility. 3MC‐participants received 3 months’ supply of ART in community ART groups (CAGs). 6MC‐participants received 6 months’ supply of ART in CAGs or at community distribution points. ART: antiretroviral treatment; IQR: interquartile range; WHO: World Health Organization.

**Figure 1:**
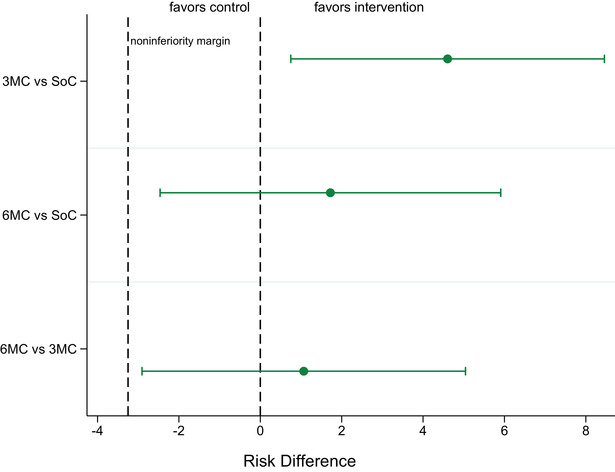
**Arm comparisons of the primary outcome (retention in antiretroviral treatment care)**. Effect measures are risk differences with 95% confidence intervals. SoC‐participants received three‐monthly dispensing of ART at the facility. 3MC‐participants received three months’ supply of ART in community ART groups (CAGs). 6MC‐participants received 6 months’ supply of ART in CAGs or at community distribution points.

After 12 months, retention was similar in all arms, 198 of 212 (93.4%), 123 of 128 (96.1%) and 248 of 259 (95.8%) in SoC, 3MC and 6MC, respectively (Table 2). In regression analyses adjusted for randomization variables and trial, retention in 3MC was superior versus SoC, adjusted risk difference (aRD) = 4.6% (95% CI: 0.7−8.5%) and retention in 6MC was non‐inferior versus SoC, aRD = 1.7% (95% CI: −2.5 to 5.9%) (Figure 1). 6MC was also non‐inferior versus 3MC. Few participants transitioned off the intervention arms due to requiring increased frequency of ART dispensing; 0.8% and 0.8% in 3MC and 6MC, respectively (Figure S1). We noted that retention amongst the small sample of participants aged <25 years was reduced and that in this age group retention in 6MC was reduced versus SoC (Tables S2 and S3). Gender was not associated with retention in this analysis, and gender was not an effect modifier.

**Table 2:** Comparison of 12‐month study outcomes between arms

	Retention in ART care (primary outcome)^a^						Viral suppression^b^						Unscheduled facility visits^c^				
			Unadjusted estimates		Adjusted estimates^d^				Unadjusted estimates		Adjusted estimates^d^			Unadjusted estimates		Adjusted estimates^d^	
	Enrolled, N	Retained, n (%)	RD (95% CI)	*p*	RD (95% CI)	*p*	Tested, n/N (%)^e^	Suppressed n (%)	RR (95% CI)	*p*	RR (95% CI)	*p*	No., mean (SD)^f^	IRR (95% CI)	*p*	IRR (95% CI)	*P*
**SoC**	212	198 (93.4)	Ref	–	Ref	–	143/198 (72.2)	142 (99.3)	Ref	–	Ref	–	0.28 (0.83)	Ref	–	Ref	–
**3MC**	128	123 (96.1)	2.9% (‐1.8 to 7.5%)	0.23	4.6% (0.7 to 8.5%)	0.02	72/122 (59.0)	71 (98.6)	0.99 (0.97–1.02)	0.73	0.98 (0.92‐1.03)	0.41	0.13 (0.49)	0.51 (0.13–1.92)	0.32	0.53 (0.16‐1.80)	0.31
**6MC**	259	248 (95.8)	2.3% (‐1.5 to 6.1%)	0.24	1.7% (‐2.5 to 5.9%)	0.42	103/243 (42.4)	101 (98.1)	0.99 (0.96–1.01)	0.33	0.98 (0.95‐1.00)	0.10	0.25 (0.67)	0.84 (0.24–2.92)	0.79	0.82 (0.25‐2.79)	0.76
**6MC (vs 3MC)**	259	248 (95.8)	‐0.6% (95% CI: −4.5 3.3%)	0.77	1.1% (‐2.9 to 5.0%)	0.60	103/243 (42.4)	101 (98.1)	0.99 (0.96–1.03)	0.64	1.0 (0.94–1.07)	0.96	0.25 (0.67)	1.55 (0.56–4.22)	0.40	1.65 (0.58‐4.74)	0.35

^a^ Risk differences were estimated using binomial population‐averaged generalized estimating equations using an exchangeable correlation structure stratified by trial, specifying for clustering by facility, using robust standard errors, and using a small cluster size variance correction. The measured intracluster correlation coefficient for retention was <0.001.

^b^ Risk ratios were estimated using log‐binomial population‐averaged generalized estimating equations using an exchangeable correlation structure stratified by trial, specifying for clustering by facility, using robust standard errors, and using a small cluster size variance correction.

^c^ Population‐averaged Poisson regression models were used to estimate incidence rate ratio's stratified by trial, specified for clustering by facility and using robust standard errors.

^d^ Adjusted estimates were adjusted for primary healthcare clinic/hospital‐based facility and rural/urban geolocation.

^e^ Those tested for viral load /those eligible for viral load testing at 12 months. Those eligible for viral load testing were enrolled participants less those who died, were lost to‐follow up or who had transferred‐out prior to 12 months after enrolment.

^f^ Number of unscheduled facility visits between months 0 to 12 of study.

SoC‐participants received three‐monthly dispensing of ART at the facility. 3MC‐participants received three months’ supply of ART in community ART groups (CAGs). 6MC‐participants received 6 months’ supply of ART in CAGs or at community distribution points. ART, antiretroviral therapy; CI, confidence interval; IRR, incidence rate ratio; RD, risk difference; Ref, reference category; RR, risk ratio.

The additional analyses using the “two‐stage” approach for the primary outcome showed similar results to the “one‐stage” approach, with heterogeneity being low. Estimated pooled RDs were 2.9% (95% CI: −1.0 to 6.8%) for 3MC versus SoC (*I* ^2^ = 0%; *p* = 0.84); and pooled RD = 2.6% (95% CI: −2.1 to 7.2%) for 6MC versus SoC (*I* ^2^ = 33%; *p* = 0.22) (Figures S2 and S3).

VL result availability at 12 months varied dramatically between districts (7‐93%) and sites (0%‐100%). Amongst those eligible for VL testing, 72.2, 59.0 and 42.4% had available VL results in SoC, 3MC and 6MC, respectively. Amongst these, VS was high and similar by arm, 99.3, 98.6 and 98.1% in SoC, 3MC and 6MC, respectively. Regression analyses confirmed that VS was similar between arms (Table 2). Differences in VS by age category were not apparent (Table S4).

Participants in all arms had few unscheduled facility visits between months 0 and 12 with little variation between arms. In regression analyses, intervention arms did not increased incidence of unscheduled facility visits (Table 2).

In this analysis of pooled data from two CRTs, including stable ART clients receiving ART for 6–12 months, retention was non‐inferior amongst participants receiving three‐ and six‐monthly community‐based MMD with single annual clinical visits for those aged ≥25 years. VS was similar, and unscheduled facility visits were not increased, which is reassuring as facility visits increase the risk of exposure to SARS‐CoV‐2. This suggests that eligibility for community‐based DSD models incorporating MMD may be safely extended to include newly stable ART clients in southern Africa to allow greater numbers of people to benefit from these models, which are also particularly relevant in the COVID‐19 era.

Strengths of our study include the randomized design that included 60 facilities in eight high HIV‐prevalence districts of southern Africa. Study limitations include the relatively small sample size that resulted in reduced power and limited precision of effect measures. Although study power was reduced, we did not increase the non‐inferiority margin compared to the original trials (in order to increase power) as we did not want to jeopardize the relatively strict criterion for non‐inferiority as defined by the original trials. The sample of participants aged <25 years was particularly small, thus, conclusions regarding this age group could not be drawn. Studies including larger sample sizes of this age group need to be conducted to ascertain if overall results are generalizable to this group. VL result availability was lower in the intervention arms; however, this was likely heavily influenced by highly variable VL testing infrastructure at different sites and districts of the study areas, reflecting differing public VL testing scale‐up that occurred during the study period. Further research in areas with good access to VL testing services should be conducted to establish if VL completion rates for out‐of‐facility models are acceptable amongst newly stable ART clients. In addition, outcomes beyond 12 months after enrolment were not measured. Further studies, including larger sample sizes and having longer participant follow‐up durations, should be conducted to validate study findings.

## 4 CONCLUSIONS

Amongst newly stable ART clients receiving ART for 6–12 months, community‐based DSD models incorporating three‐ and six‐monthly ART refills with single annual clinical visits were at least non‐inferior to standard three‐monthly facility‐based care amongst those aged ≥25 years. These models should be considered for scaling in light of both the COVID‐19 pandemic and to allow more people to benefit from these patient‐centred models. Few participants aged <25 years were included, and further research to ascertain if community‐based DSD models effectively retain newly stable ART clients in this age group should be conducted. Further research is also needed to assess whether community‐based DSD models are suitable for those who have initiated ART within 6 months.

## COMPETING INTERESTS

The authors declare that they have no conflict of interest.

## AUTHORS’ CONTRIBUTIONS

GF, NNM, AT, BBT, CL, CC, AG designed the research study. GF, NNM, AT, BBT, TK, KM, CC, AG performed the research. GF and TM analyzed the data. GF wrote the paper. All authors have read and approved the final manuscript.

## FUNDING

President's Emergency Plan for AIDS Relief through the United States Agency for International Development EQUIP mechanism (Grant number AID‐OAA‐A‐15‐00070).

## Supporting information


**Table S1**. Brief description of the model of care for each arm of the trials
**Table S2**. Individual‐level factors associated with participant retention in ART care 12 months after enrolment (primary outcome)
**Table S3**. Arm comparison of retention in care after 12 months stratified by age category
**Table S4**. Arm comparison of viral suppression after 12 months stratified by age category
**Figure S1**. Study flow diagram
**Figure S2**. Forest plot of estimated pooled risk difference of retention in ART care at 12 months for arm 3MC vs. SoC
**Figure S3**. Forest plot of estimated pooled risk difference of retention in ART care at 12 months for arm 6MC vs. SoC

## References

[1] Traub AM , Ifafore‐Calfee T , Frymus D , Phelps BR . Multimonth dispensing of antiretroviral therapy for HIV. Lancet HIV. 2020; 7(7):e457–e8. 32621873 10.1016/S2352-3018(20)30169-7

[2] Eshun‐Wilson I , Mukumbwa‐Mwenechanya M , Kim HY , Zannolini A , Mwamba CP , Dowdy D , et al. Differentiated care preferences of stable patients on antiretroviral therapy in Zambia: a discrete choice experiment. J Acquir Immune Defic Syndr. 2019; 81(5):540–6. 31021988 10.1097/QAI.0000000000002070 PMC6625870

[3] Keene CM , Zokufa N , Venables EC , Wilkinson L , Hoffman R , Cassidy T , et al. ‘Only twice a year’: a qualitative exploration of 6‐month antiretroviral treatment refills in adherence clubs for people living with HIV in Khayelitsha, South Africa. BMJ Open. 2020; 10(7):e037545. 10.1136/bmjopen-2020-037545 PMC7348319 32641338

[4] Wilkinson L , Grimsrud A . The time is now: expedited HIV differentiated service delivery during the COVID‐19 pandemic. J Int AIDS Soc. 2020; 23(5):e25503. 32378345 10.1002/jia2.25503 PMC7203569

[5] Traub AM , Ifafore‐Calfee T , Phelps BR . Multimonth dispensing of antiretroviral therapy protects the most vulnerable from 2 pandemics at once. Glob Health Sci Pract. 2020; 8(2):176–7. 32606089 10.9745/GHSP-D-20-00160 PMC7326512

[6] Tukei BB , Fatti G , Tiam A , Ngorima‐Mabhena N , Tukei VJ , Tshabalala I , et al. Twelve‐month outcomes of community‐based differentiated models of multimonth dispensing of ART among stable HIV‐infected adults in Lesotho: a cluster‐randomized noninferiority trial. J Acquir Immune Defic Syndr. 2020; 85(3):280–91. 32665460 10.1097/QAI.0000000000002439

[7] Cassidy T , Grimsrud A , Keene C , Lebelo K , Hayes H , Orrell C , et al. Twenty‐four‐month outcomes from a cluster‐randomized controlled trial of extending antiretroviral therapy refills in ART adherence clubs. J Int AIDS Soc. 2020; 23(12):e25649. 33340284 10.1002/jia2.25649 PMC7749539

[8] Fatti G , Ngorima‐Mabhena N , Mothibi E , Muzenda T , Choto R , Kasu T , et al. Outcomes of three‐ versus six‐monthly dispensing of antiretroviral treatment (ART) for stable HIV patients in community ART refill groups: a cluster‐randomized trial in Zimbabwe. J Acquir Immune Defic Syndr. 2020; 84(2):162–72. 32097252 10.1097/QAI.0000000000002333 PMC7172979

[9] Prust ML , Banda CK , Nyirenda R , Chimbwandira F , Kalua T , Jahn A , et al. Multi‐month prescriptions, fast‐track refills, and community ART groups: results from a process evaluation in Malawi on using differentiated models of care to achieve national HIV treatment goals. J Int AIDS Soc. 2017; 20(Suppl 4):21650. 28770594 10.7448/IAS.20.5.21650 PMC5577715

[10] Prust ML , Banda CK , Callahan K , Nyirenda R , Chimbwandira F , Kalua T , et al. Patient and health worker experiences of differentiated models of care for stable HIV patients in Malawi: a qualitative study. PLoS One. 2018; 13(7):e0196498. 30024874 10.1371/journal.pone.0196498 PMC6053133

[11] Mody A , Roy M , Sikombe K , Savory T , Holmes C , Bolton‐Moore C , et al. Improved retention with 6‐month clinic return intervals for stable human immunodeficiency virus‐infected patients in Zambia. Clin Infect Dis. 2018; 66(2):237–43. 29020295 10.1093/cid/cix756 PMC5850531

[12] Ross J , Murenzi G , Hill S , Remera E , Ingabire C , Umwiza F , et al. Reducing time to differentiated service delivery for newly diagnosed people living with HIV in Kigali, Rwanda: study protocol for a pilot, unblinded, randomised controlled study. BMJ Open. 2021; 11(4):e047443. 10.1136/bmjopen-2020-047443 PMC8074553 33895720

[13] International AIDS Society. Differentiated Service Delivery. 2021. https://differentiatedservicedelivery.org/Portals/0/adam/Content/jcdklT8RzEqirRdlckAjbQ/File/1‐Time%20to%20DSD%20Eligibility%20D5.pdf

[14] Hoffman RM , Balakasi K , Bardon AR , Siwale Z , Hubbard J , Kakwesa G , et al. Eligibility for differentiated models of HIV treatment service delivery: an estimate from Malawi and Zambia. AIDS. 2020; 34(3):475–9. 31764076 10.1097/QAD.0000000000002435

[15] World Health Organization . Updated Recommendations on Service Delivery for the Treatment And Care of People Living with HIV Geneva. 2021. https://www.who.int/publications/i/item/9789240023581] 33999550

[16] Nichols BE , Cele R , Lekodeba N , Tukei B , Ngorima‐Mabhena N , Tiam A , et al. Economic evaluation of differentiated service delivery models for HIV treatment in Lesotho: costs to providers and patients. J Int AIDS Soc. 2021; 24(4):e25692. 33838012 10.1002/jia2.25692 PMC8035675

[17] Fatti G , Ngorima‐Mabhena N , Chirowa F , Chirwa B , Takarinda K , Tafuma TA , et al. The effectiveness and cost‐effectiveness of 3‐ vs. 6‐monthly dispensing of antiretroviral treatment (ART) for stable HIV patients in community ART‐refill groups in Zimbabwe: study protocol for a pragmatic, cluster‐randomized trial. Trials. 2018; 19(1):79. 29378662 10.1186/s13063-018-2469-y PMC5789674

[18] Faturiyele IO , Appolinare T , Ngorima‐Mabhena N , Fatti G , Tshabalala I , Tukei VJ , et al. Outcomes of community‐based differentiated models of multi‐month dispensing of antiretroviral medication among stable HIV‐infected patients in Lesotho: a cluster randomised non‐inferiority trial protocol. BMC Public Health. 2018; 18(1):1069. 30157896 10.1186/s12889-018-5961-0 PMC6116392

[19] World Health Organization . Consolidated guidelines on the use of antiretroviral drugs for treating and preventing HIV infection recommendations for a public health approach (Second edition). Geneva, Switzerland: WHO; 2016. http://apps.who.int/iris/bitstream/10665/208825/1/9789241549684_eng.pdf?ua=1] 27466667

[20] Stricker SM , Fox KA , Baggaley R , Negussie E , de Pee S , Grede N , et al. Retention in care and adherence to ART are critical elements of HIV care interventions. AIDS Behav. 2014; 18(5):465–75. 10.1007/s10461-013-0598-6 24292251

[21] Tierney JF , Vale C , Riley R , Smith CT , Stewart L , Clarke M , et al. Individual participant data (IPD) meta‐analyses of randomised controlled trials: guidance on their use. PLoS Med. 2015; 12(7):e1001855. 26196287 10.1371/journal.pmed.1001855 PMC4510878

[22] Stewart GB , Altman DG , Askie LM , Duley L , Simmonds MC , Stewart LA . Statistical analysis of individual participant data meta‐analyses: a comparison of methods and recommendations for practice. PLoS One. 2012; 7(10):e46042. 23056232 10.1371/journal.pone.0046042 PMC3463584

[23] Stijnen T , Hamza TH , Özdemir P . Random effects meta‐analysis of event outcome in the framework of the generalized linear mixed model with applications in sparse data. Stat Med. 2010; 29(29):3046–67. 20827667 10.1002/sim.4040

[24] Thomas D , Radji S , Benedetti A . Systematic review of methods for individual patient data meta‐ analysis with binary outcomes. BMC Med Res Methodol. 2014; 14(1):79. 24943877 10.1186/1471-2288-14-79 PMC4074845

