# Supplementary material for: Community‐based differentiated service delivery models incorporating multi‐month dispensing of antiretroviral treatment for newly stable people living with HIV receiving single annual clinical visits: a pooled analysis of two cluster‐randomized trials in southern Africa
Source: J Int AIDS Soc. 2021 Oct 28;24(Suppl 6):e25819. doi: 10.1002/jia2.25819 (PMC8554219; doi:10.1002/jia2.25819)
Supplement: Supplementary file 1 — Table S1. Brief description of the model of care for each arm of the trials Table S2. Individual‐level factors associated with participant retention in ART care 12 months after enrolment (primary outcome) Table S3. Arm comparison of retention in care after 12 months stratified by age category Table S4. Arm comparison of viral suppression after 12 months stratified by age category Figure S1. Study flow diagram Figure S2. Forest plot of estimated pooled risk difference of retention in ART care at 12 months for arm 3MC vs. SoC Figure S3. Forest plot of estimated pooled risk difference of retention in ART care at 12 months for arm 6MC vs. SoC [file JIA2-24-e25819-s001.docx]

**Supplementary Material**

**Supplementary Table 1: Brief description of the model of care for each arm of the trials**

|  | **Standard of care (SoC)** | **Arm 3MC** | **Arm 6MC** |
| --- | --- | --- | --- |
| **Frequency of ART refills** | 3 monthly | 3 monthly | 6 monthly |
| **Frequency of clinical consultations** | 3 monthly | 12 monthly | 12 monthly |
| **Location of ART refills** | Facility | Community ART group | Community ART group (Zimbabwe).  Community distribution point (Lesotho) |
| **Frequency of viral load testing** | 12 monthly | 12 monthly | 12 monthly |
| **No. of people in the model per country^†^** | 1898 Lesotho  1919 Zimbabwe | 1558 Lesotho  1335 Zimbabwe | 1880 Lesotho  1546 Zimbabwe |
| **No. of sites** | 10 Lesotho  10 Zimbabwe | 10 Lesotho  10 Zimbabwe | 10 Lesotho  10 Zimbabwe |

^†^ Total number of participants included in the original trials irrespective of time since antiretroviral treatment initiation

SoC-participants received three-monthly dispensing of ART at the facility. 3MC-participants received three months’ supply of ART in community ART groups (CAGs). 6MC-participants received 6 months’ supply of ART in CAGs (Zimbabwe) or at community distribution points (Lesotho).

**Supplementary text: Further details regarding the interventions (arms 3MC and 6MC)**

Community ART groups (CAGs) consisted of 6-12 people, with participants living in similar geographic areas who met at a community venue. For the 3MC arm, a single alternating CAG representative collected ART from the facility on a three-monthly basis and distributed the medicines to other CAG members at the CAG meeting. For the 6MC arm in Zimbabwe, a single CAG representative collected six-monthly ART supplies from the facility 6 months after enrolment and distributed it to all CAG members. In Lesotho, 6MC participants received a six-monthly ART supply at community distribution points six months after enrolment on an individual provider-client basis from a healthcare worker certified to dispense ART to stable clients. Tracking of participants who disengaged from care was as per routine site procedures.

All enrolled participants provided informed consent for trial participation.

**Supplementary text: “One-stage” individual participant data meta-analyses**

For retention in ART care (primary outcome), risk differences were estimated using binomial population-averaged generalized estimating equations (GEE) using an exchangeable correlation structure, specifying for clustering by facility, using robust standard errors, and using a small cluster size variance correction [1,2]. Log-binomial population-averaged GEE models were used to compare viral suppression (secondary outcome) using an exchangeable correlation structure, specifying for clustering by facility, using robust standard errors, and using a small cluster size variance correction. Population-averaged Poisson models were used to compare unscheduled facility visits, specified for clustering as above and using robust standard errors. Regression models were stratified by trial. Adjusted analyses were conducted controlling for variables used for the stratified randomization (urban/rural location and primary healthcare/hospital-based facility). Separate models were also constructed for the primary outcome adjusted for gender and baseline age. Analyses were conducted using Stata^TM^ version 16.1.

4800 Enrolled participants in Zimbabwe trial (30 clusters)

5336 Enrolled participants in Lesotho trial (30 clusters)

3426 Arm 6MC (20 clusters)

3817 Arm SoC (control); (20 clusters)

2893 Arm 3MC (20 clusters)

2765 Enrolled > 12 months after ART initiation excluded

3605 Enrolled > 12 months after ART initiation excluded

3167 Enrolled > 12 months after ART initiation excluded

212 included in SoC (control)

cluster size: median 10 (IQR: 6.5–13.5). CV: 0.49

259 included in 6MC

cluster size: median 10.5 (IQR: 8–18.5). CV: 0.52

128 included in 3MC

cluster size: median 5 (IQR: 2–9). CV: 0.90

12 months after enrolment:

241 remained in study model of care

0 died

11 lost to follow-up

5 transferred out

2 transitioned off study arm

12 months after enrolment:

121 remained in study model of care

0 died

5 lost to follow-up

1 transferred out

1 transitioned off study arm

12 months after enrolment:

197 remained in study model of care

1 died

13 lost to follow-up

0 transferred out

1 transitioned off study arm

**Supplementary Figure 1: Study flow diagram**

ART: antiretroviral treatment; CV: coefficient of variation of cluster size; IQR: Interquartile range

**Supplementary Table 2: Individual-level factors associated with participant retention in ART care 12 months after enrolment (primary outcome)**

| **Baseline variable** | **Unadjusted estimates** | | **Adjusted estimates** | |
| --- | --- | --- | --- | --- |
|  | **RD (95% CI)** | ***P*** | **RD (95% CI)** | ***P*** |
| **Arm** |  |  |  |  |
| **SoC** | Ref | – | Ref | – |
| **3MC** | 2.9 (-1.8 to 7.5) | 0.23 | 1.6 (-2.9 to 6.2) | 0.49 |
| **6MC** | 2.3 (-1.5 to 6.1) | 0.24 | 2.7 (-0.6 to 6.0) | 0.11 |
| **Age category** |  |  |  |  |
| **18–24 years** | -13.6 (-26.0 to -1.3) | 0.03 | -14.3 (-27.0 to -1.7) | 0.026 |
| **25–49 years** | Ref | – | Ref | – |
| **≥ 50 years** | 3.6 (0.5 to 6.6) | 0.02 | 2.4 (-0.6 to 5.5) | 0.124 |
| **Gender** |  |  |  |  |
| **Female** | Ref | – |  |  |
| **Male** | 0.6 (-0.3 to 4.0) | 0.72 |  |  |

SoC-participants received three-monthly dispensing of ART at the facility. 3MC-participants received three months’ supply of ART in community ART groups (CAGs). 6MC-participants received 6 months’ supply of ART in CAGs or at community distribution points. RD: risk difference; CI: confidence interval; WHO: World Health Organization; Ref: reference category

**Supplementary table 3: Arm comparison of retention in care after 12 months stratified by age category**

|  | **18–24 years** | | | | **25–49 years** | | | | **≥50 years** | | | |
| --- | --- | --- | --- | --- | --- | --- | --- | --- | --- | --- | --- | --- |
| **Arm** | **Enrolled, N** | **Retained, n (%)** | **RD (95% CI)** | **P-value** | **Enrolled, N** | **Retained, n (%)** | **RD (95% CI)** | **P-value** | **Enrolled, N** | **Retained, n (%)** | **RD (95% CI)** | **P-value** |
| **SoC** | 15 | 14 (93.3) | Ref | - | 151 | 138 (91.4) | Ref | - | 46 | 46 (100) | Ref | - |
| **3MC** | 4 | 4 (100) | 0 |  | 91 | 87 (95.6) | 4.2 (-2.3 to 10.8) | 0.21 | 33 | 32 (97.0) | -1.4 (-8.0 to 5.1) | 0.67 |
| **6MC** | 13 | 8 (61.5) | -20.0 (-34.0 to -5.9) | 0.005 | 189 | 184 (97.4) | 5.9 (0.9 to 11.0) | 0.021 | 57 | 56 (98.3) | 0 | - |

SoC-participants received three-monthly dispensing of ART at the facility. 3MC-participants received three months’ supply of ART in community ART groups (CAGs). 6MC-participants received 6 months’ supply of ART in CAGs or at community distribution points. RD: risk difference; CI: confidence interval; Ref: Reference category

**Supplementary table 4: Arm comparison of viral suppression after 12 months stratified by age category**

|  | **18–24 years** | | | | **25–49 years** | | | | **≥50 years** | | | |
| --- | --- | --- | --- | --- | --- | --- | --- | --- | --- | --- | --- | --- |
| **Arm** | **Tested, n/N (%)** | **Suppressed** | **RR (95% CI)** | **P-value** | **Tested, n/N (%)** | **Suppressed** | **RR (95% CI)** | **P-value** | **Tested, n/N (%)** | **Suppressed** | **RR (95% CI)** | **P-value** |
| **SoC** | 11/14 (78.6) | 11 (100) | Ref | - | 100/139 (71.9) | 99/100 (99) | Ref | - | 32/46 (69.6) | 32/32 (100) | Ref | - |
| **3MC** | 0/4 (0) | - | 1 |  | 55/86 (64.0) | 55/55 (100) | 1 |  | 17/32 (53.1) | 16/17 (94.1) | 1 | - |
| **6MC** | 5/8 (65.5) | 5/5 (100) | 1 |  | 77/179 (43) | 75/77 (97.4) | 0.98 (0.95–1.02) | 0.37 | 21/56 (37.5) | 21/21 (100) | 1 | - |

SoC-participants received three-monthly dispensing of ART at the facility. 3MC-participants received three months’ supply of ART in community ART groups (CAGs). 6MC-participants received 6 months’ supply of ART in CAGs or at community distribution points. RR: risk ratio; Ref: reference category

**Supplementary Figure 2: Forest plot of estimated pooled risk difference of retention in ART care at 12 months for arm 3MC vs. SoC.**

**Supplementary Figure 3: Forest plot of estimated pooled risk difference of retention in ART care at 12 months for arm 6MC vs. SoC.**

**References for the supplementary material**

1. Huang S, Fiero MH, Bell ML. Generalized estimating equations in cluster randomized trials with a small number of clusters: Review of practice and simulation study. Clinical Trials. 2016;13(4):445-9.

2. Pedroza C, Thanh Truong VT. Performance of models for estimating absolute risk difference in multicenter trials with binary outcome. BMC Med Res Methodol. 2016;16(1):113.
